# Supplementary material for: A high-resolution mRNA expression time course of embryonic development in zebrafish
Source: eLife. 2017 Nov 16;6:e30860. doi: 10.7554/eLife.30860 (PMC5690287; doi:10.7554/eLife.30860)
Supplement: Supplementary file 6. [file elife-30860-supp6.zip › biolayout-clusters-files/Cluster213-genes.html]

Cluster213


# Cluster213: Genes

| Ensembl ID | Gene Name | Chr | Start | End | Biotype |
| --- | --- | --- | --- | --- | --- |
| ENSDARG00000100573 | ckap2l | 5 | 13049386 | 13060857 | protein\_coding |
| ENSDARG00000074796 | dbf4 | 16 | 43235746 | 43246731 | protein\_coding |
| ENSDARG00000070787 | jupa | 11 | 11735954 | 11797611 | protein\_coding |
| ENSDARG00000004049 | marcksa | 20 | 3298762 | 3302235 | protein\_coding |
| ENSDARG00000038909 | msl2a | 2 | 25468864 | 25473775 | protein\_coding |
| ENSDARG00000061527 | nxt2 | 14 | 28153481 | 28167681 | protein\_coding |
| ENSDARG00000092583 | si:dkey-16p19.5 | 4 | 40685264 | 40695165 | protein\_coding |
